# Operando X‐Ray Tomoscopy of Laser Beam Welding

**DOI:** 10.1002/advs.202413108

**Published:** 2025-01-13

**Authors:** Paul Hans Kamm, Stephan Börner, Tillmann Robert Neu, Christian Matthias Schlepütz, Dirk Dittrich, John Banhart, Francisco García‐Moreno

**Affiliations:** ^1^ Institute of Applied Materials Helmholtz‐Zentrum Berlin für Materialien und Energie Hahn‐Meitner‐Platz 1 14109 Berlin Germany; ^2^ Institute of Materials Science and Technology Technische Universität Berlin Hardenbergstraße 36 10623 Berlin Germany; ^3^ Fraunhofer Institute for Material and Beam Technology IWS Winterbergstraße 28 01277 Dresden Germany; ^4^ Swiss Light Source Paul Scherrer Institute Forschungsstraße 111 5232 Villigen PSI Switzerland

**Keywords:** dynamic beam modulation, keyhole, laser welding, pore formation, X‐ray tomoscopy

## Abstract

The phenomena occurring in a weld seam during advancement of a laser beam over a metallic component are still under dispute. The occurrence and evolution of porosity and the occasional blowout of melt need to be understood. Here, a recently developed X‐ray tomoscopy setup is applied, providing one hundred 3D images per second to capture the temporal evolution of the melt pool in an AlSi9Cu3(Fe) die‐casting while a laser beam advances. The number of pores, their size, shape and distribution are quantified with 10 ms time resolution and reflect a complex dynamic pattern. Apart from conventional welding, a variant involving a dynamic beam modulation superimposed onto the linear motion is studied. Reductions of porosity and surface roughness are observed and explained by increased pore mobility and stepwise degassing as the beam repeatedly cuts through pores. The keyhole formed in the melt pool integrated over 10 ms is represented in 3D.

## Graphical abstract

An advanced way of laser beam guidance in the welding of die‐cast aluminum by beam modulation is investigated in 3D over time using X‐ray tomoscopy. New insights into the interaction of the keyhole with the forming porosity are gained. These dynamic changes are quantified with a temporal resolution of 10 ms and a reduction in porosity and surface roughness is observed.

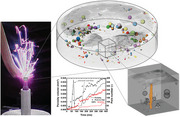

## 1 Introduction

The amount of aluminum and aluminum alloys for lightweight applications is steadily increasing, especially due to the increasing electrification of vehicles.^[^ ^1^ ^]^ More intensive cooperation between aluminum producers, job‐shop operators and end manufacturers ahead could open further potential for the use of aluminum alloys. In that sense, advanced joining technologies such as laser beam welding are the connecting link between them.^[^ ^2^, ^3^ ^]^

Laser beam keyhole welding is a fusion process in which the required energy is supplied by a laser. The working principle is that the energy of the laser beam focused onto a workpiece is absorbed near the surface, which induces local melting. When the vaporization temperature of the material is reached, the out‐flowing metal vapor creates a capillary (keyhole) in the molten pool.^[^ ^4^ ^]^ A pressure of several thousand Pa prevailing in the vapor capillary is in equilibrium with the surface tension and the hydrostatic and dynamic pressure of the melt. This is why the capillary is maintained even for a superimposed relative movement. A weld seam is created by such a relative movement between the laser beam and the workpieces, after which solidification sets in and joins the pieces.^[^ ^4^ ^]^

One challenge in laser beam welding applications is the avoidance of pores, especially with aluminum materials. Several mechanisms can lead to those irregularities. Various investigations in the literature show that the main causes for pore formation are the increased solubility of hydrogen and other gases in the molten material, contaminations, or inclusions, as well as keyhole phenomena like strong fluctuations and constriction of the keyhole during the process.^[^ ^5^, ^6^, ^7^, ^8^, ^9^ ^]^ Aluminum alloy die‐cast pieces are difficult or almost impossible to weld due to the high solubility of hydrogen and pressurized gas in cavities. This can lead to strong pore formation and melt ejections during welding and consequently to inferior local properties around the welding zone such as low stiffness, ductility and fatigue strength.^[^ ^1^, ^10^ ^]^ One potential solution to counteract these issues is the usage of beam oscillation—as a superimposed movement of the beam during the feed motion—to stabilize the keyhole as well as the welding process.^[^ ^1^, ^7^, ^11^, ^12^ ^]^

The interactions inside such molten weld seams are hard to observe in situ. 2D time‐resolved X‐ray radioscopy up to 50 000 fps was applied successfully in the past to explore the welding process.^[^ ^7^, ^13^, ^14^, ^15^, ^16^, ^17^, ^18^, ^19^ ^]^ With its help, it was possible to study fast phenomena inside the complex weld zone. However, the knowledge gained in these studies suffers from the usual limitations of 2D radioscopy, namely application only to thin samples with lateral thermal constraints and overlap of information in the beam direction, thus avoiding proper time‐resolved quantitative 3D analyses. X‐ray tomography avoids the ambiguities and interpretations of radiography caused by the superposition of features in one direction and the associated loss of information. “Tomoscopy” is a synonym for time‐resolved tomography, which sometimes is also called 4D tomography.^[^ ^20^ ^]^ In the field of materials science, it has proven to be a method that provides unprecedented insights into dynamic processes on the micrometer scale.^[^ ^21^ ^]^ Some applications are the observation of phase transformations: For example, the formation of pores during the production of metallic foams, the observation of the solidification process in metals, the formation of structures during freeze casting or the investigation of the behaviour of materials under stress, e.g., the propagation of cracks.^[^ ^20^, ^21^, ^22^, ^23^, ^24^, ^25^ ^]^ In the context of additive manufacturing, tomoscopy has also been used to monitor the formation and evolution of defects during the laser powder bed fusion process, facilitating the optimization of printing parameters to improve material properties and structural integrity.^[^ ^26^ ^]^ The limits of the method are primarily the achievable rates using conventional techniques and the associated loads on the sample, which limit its final dimensions. However, the development of modern synchrotron sources and detectors allows continuous improvements in the spatial resolution and the time span to be acquired.

To study the welding process of aluminum sheets and explore fast phenomena related to the process quantitatively, time‐resolved and in 3D, the newly developed X‐ray tomoscopy setup was employed in this work at the Swiss Light Source.^[^ ^21^ ^]^ It allows for studying the formation and temporal evolution of the weld melt pool, as well as porosity formation and evolution during melting and solidification. The advantages over conventional X‐ray radioscopy are the determination of the true location, for example in the width of the welding seam, and the shape and orientation of the porosity and keyhole, especially for non‐linear beam guidance. Through in‐situ X‐ray analysis, the investigation of the underlying stabilization mechanisms of laser beam welding with an oscillating beam is possible and enables a deeper understanding of the process and the associated pore formation.

## 2 Results and Discussion

**Figure 1:**
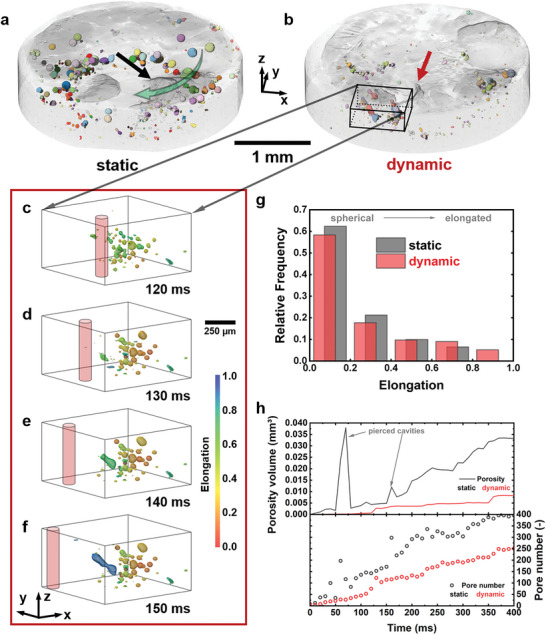
Rendered tomograms showing the porosity distribution represented by separated, colored pores in the circular weld seam of cylindrical die‐cast AlSi9Cu3(Fe) samples, welded a) with a linear beam feed (static) and b) with a dynamic beam oscillating with a frequency of 1 kHz, both in the final state after welding (*t* = 400 ms). Welding follows the green arrow (a). The black (a) and red (b) arrows point at irregularities on the seam surface. c–f) Magnification of the inset in b) showing time‐resolved the formation, growth and disappearance of bubbles and the formation of elongated pores at the liquid‐solid interface between 120 ms and 150 ms. The keyhole is schematically represented by a light red cylinder and the bubbles are color‐coded according to their elongation. g) Distributions of pore elongation in the final state. h) Temporal evolution of porosity volume and pore number in samples shown in a) and b).

Rendered images of X‐ray tomograms extracted from the end of the tomoscopic series of the welding process of cylindrical samples are shown in **Figure** **1**. The alloy is transparent, while the porosity is represented by colors. Mostly spherical pores are seen within the circular weld seam. There is a clear reduction of the amount, volume fraction and size of the pores when going from the linear beam feed without oscillation, also referred to as “static” from now on (Figure 1a), to the dynamic, oscillating condition (Figure 1b). The region marked with a black cuboid in Figure 1b in the sample welded with an oscillating beam is resolved in further detail and time in Figure 1c–f. In Figure 1c pores in the melt pool directly around the keyhole (position represented by a red cylinder) can be seen, of which some can escape or coalesce with others and can no longer be observed 10 ms later, see Figure 1d. Figure 1e,f shows the sporadic formation of elongated pores near the solid‐liquid interface, which develop in tens of milliseconds and are oriented upwards at an angle of ∼45 ° to the laser beam. Figure 1g shows that most of the pores have a small elongation between 0 and 0.2. The relative frequency of elongated pores (0.6–1) in the final state after welding is higher for the sample welded under dynamic beam guidance, while the frequency of round pores (0–0.4) is lower compared to the static condition.

From quantitative image analysis, the temporal evolution of porosity volume and pore number for both welding conditions is obtained. Figure 1h shows a reduction in the number of pores by 38 % in the sample produced with beam oscillation compared to the static condition. The number of pores increases within the first 100 ms at an average rate of ∼0.5 (ms)^−1^ and later at ∼0.67 (ms)^−1^ for the sample treated with an oscillating beam, while the rate for the static case is higher and more irregular. The total number of pores given in Figure 1h comprises pores in already solidified areas as well as bubbles in the molten region around the keyhole. The latter can vary much more and more rapidly, both in terms of sizes and numbers, which leads to an irregular increase of the curves in Figure 1h. Oscillation reduces pore volume by 75 % and gives rise to a slower and more continuous growth of pore volume, thus avoiding the sudden steps seen for the static beam.

**Figure 2:**
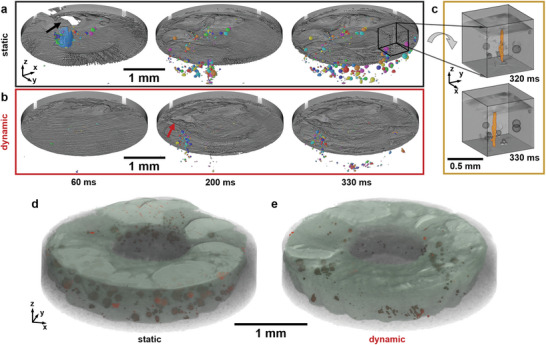
Rendered images of the upper sample surface observed from inside the sample and porosity distribution represented by separated, coloured pores in the circular weld seam at selected times recorded operando during laser welding: with a a) static and b) dynamic beam. Same sense of beam feed as in Figure 1. The black arrow in a) marks melt blowout, the red arrow in b) a seam interruption at the sample surface. c) Magnification of the inset in a) viewed from above, showing in detail the evolution of the keyhole and bubbles in a time increment of 10 ms. d,e) Rendered tomograms of the isolated weld seam (transparent green) and pores (red) in the final state after welding for the d) static and e) dynamic condition.

**Figure** **2**a,b shows time‐resolved rendered tomograms of the sample surface (in grey) and the evolution of porosity in the weld seam beneath the surface represented by colored pores for both conditions and three selected times. The beneficial effect of beam oscillation can be seen very clearly. Bubbles in the molten region close to the keyhole change in their size and shape and occasionally coalesce. In the static case, a big bubble and a large hole at the surface appear already after 60 ms (Figure 2a) but recover to a certain extent as welding advances due to inflowing melt. In the dynamic case (Figure 2b), a much more homogeneous course of the weld seam can be seen over most of the surface, with fewer and smaller reinforcement of the weld bead. In Figure 2c, the temporal evolution over an interval of 10 ms, i.e., between two successive tomograms, of the region marked by a black cube in Figure 2a is shown in detail. Under the static condition a 3D representation of the keyhole integrated over 10 ms can be generated. The position and shape of the ∼0.6 mm long and ∼30μm thick keyhole (in orange) varies in time, i.e., the keyhole fluctuates, and moves with the laser beam in the welding direction, resulting in a slight blurring. Furthermore, the positions and sizes of the surrounding bubbles change very fast. They appear, coalesce, or vanish in milliseconds as can be observed in the short period shown in Figure 2c. For the oscillating beam, the recording speed of 100 tomograms per second (tps) is not sufficient to resolve the keyhole structure without blurring owing to the oscillation at 1 kHz. The welded area can be extracted as shown in transparent green in Figure 2d,e. The seams, that follow a circle with a diameter of 2 mm, vary slightly in width and show a typical weld bead on the surface. They reach their largest width of ∼800μm at the sample surface and their volumes differ only slightly with 2.5  mm^3^ for the static and 2.3  mm^3^ for the dynamic beam. Since almost all the porosity is concentrated in the weld, the same picture emerges as for the absolute porosity volume, where the porosity of the statically welded seam is about 1.45 %, that of the dynamically welded only 0.35 %.

**Figure 3:**
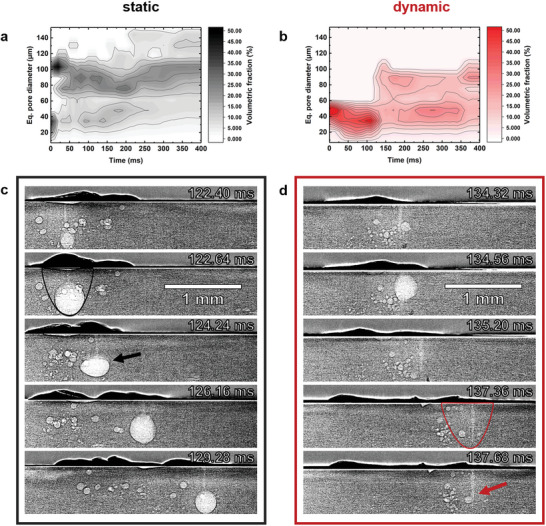
Temporal evolution of the equivalent diameter of pores formed at the circular seams recorded in situ during laser welding: a) static, b) dynamic beam. Data derived from tomoscopic images. c–d) Selected time‐resolved radiograms of the evolving keyhole and porosity of both conditions. The solid‐liquid interfaces of the molten areas are outlined in black and red.

The time dependence of the volume‐weighted equivalent pore diameter distributions is displayed in **Figure** **3**a,b. For the static beam, a bimodal equivalent pore diameter distribution of small and large pores with a pronounced volumetric fraction in the first 20 to 50 ms can be observed in Figure 3a, with populations around 30μm and 100μm. After the initial 250 ms the distribution is almost entirely determined by large pores distributed around an equivalent pore diameter of ∼100μm. On the contrary, the oscillating beam produces only pores with equivalent diameters smaller than 60μm in the first ∼120 ms (see Figure 3b). After that, additional larger pores of about 90μm form suddenly, leading again to a bimodal distribution towards the end of the experiment, but this time with markedly smaller porosity volume, as shown previously in Figure 1h.

Figure 3c,d shows radioscopic X‐ray images of the rotating samples during the measurements. They can help to understand the process in more detail due to the higher temporal resolution, although they do not provide the same depth of information as 3D volumes. Due to the short exposure time of 75μs per image, the keyhole can be observed (see e.g., red arrow in lower Figure 3d). Owing to the influence of phase contrast, also the liquid‐solid interface can be detected around the keyhole, see black and red contour lines in Figure 3c,d. The extension is similar in both conditions, with a slightly larger expansion of the melt pool behind the keyhole in the direction of movement. The evolution of the liquid‐solid interface and of the keyhole can be better observed in the supplementary material (Video S1, Supporting Information).

In the static welding condition, Figure 3c, a bubble appears due to the action of the keyhole at 122.40 ms and is inflated to a large bubble at 122.64 ms, associated with strong material blowout from the marked melt pool. The large bubble remains captured by the keyhole for longer than 7 ms and the keyhole's shape fluctuation can be recognized by the vertical position and shape change of the bubble (indicated by a black arrow). However, for the oscillating beam, large bubbles vanish quickly in less than 1 ms as shown in Figure 3d between 134.32 and 135.20 ms. The keyhole oscillations can also be observed indirectly through the relative movement of the formed bubbles that follow this motion (red arrow in Figure 3d; Video S1, Supporting Information).

In conventional welding with static laser beam guidance, porosity can be caused by the release of gas enclosed in the material like hydrogen, whose solubility in the molten aluminum is 20 times higher than in the solid state.^[^ ^10^ ^]^ Another cause for porosity are instabilities of the keyhole, when parts of the keyhole cut off and can no longer degas due to the ongoing solidification.^[^ ^27^, ^28^ ^]^ By observing the welding process by X‐ray imaging it could be found out, that a piercing of pressurized cavities that frequently occur in die‐cast alloys lead to high porosity and blow outs as well.^[^ ^1^, ^7^ ^]^ Their quantity and distribution in the base material is important. During our welding experiments, only a volume of about 10^−4^  mm^3^ of spatially resolvable cavities, distributed over a small number of locations (see Figure S1, Supporting Information), is passed over by the melt pool. The cavities can be seen in the distributions of the equivalent diameter in Figure 3a,b at the beginning in the range between 10μm and 60μm. Tomoscopy allows to determine the positions of these local gas sources with respect to the melt pool and to correlate them with increased bubble formation (see Figure S2, Supporting Information). Thereby, the volume of newly generated gas pores, as seen by the steps in the porosity in Figure 1h, is many times higher for the maximum difference between two tomograms with up to 22.5 × 10^−3^  mm^3^ for the static and 2.5 × 10^−3^  mm^3^ for the oscillating beam. When the melt pool touches a gas cavity, the pressure conditions around the capillary change immediately: A large bubble forms rapidly and molten material is ejected. In the radiographies of the statically welded specimen shown in Figure 3c, the relationship between entrapped gases and weld irregularities like blow outs and cratering is evident. Such irregularities are marked by black arrows in Figures 1 and 2 and are known from the literature.^[^ ^7^ ^]^

Bubbles formed in the melt tend to remain spherical due to the influence of surface tension, but move fast inside the molten region due to strong convection in the melt. At some point they settle at the edge of the melting line, get pinned at the liquid–solid interface and turn into spherical pores as the material around the bubbles solidifies after the keyhole and the associated molten region has advanced. The large bubble shown in Figure 3c grows to a diameter of more than 380μm and remains in place for a long time (∼11 ms until complete degassing—see Video S1, Supporting Information). The energy is introduced in a very concentrated manner, resulting in a strong temperature gradient in the melt pool. Therefore, the large bubble formed in the vicinity of the keyhole are presumably also due to the hydrogen intake of the molten aluminum alloy. Since such large bubbles only persist over one to two tomograms, this creates peaks in the porosity volume as seen in Figure 1h.

In contrast, for dynamic beam oscillation, large bubbles vanish rapidly in less than 1 ms as shown in Figure 3d, because of the stepwise degassing to the surface of the melt pool. Additionally, the pressurized cavities were pierced stepwise and smaller bubbles appeared, which are easier to degas. The positive effect from the repeated transition of the keyhole through the bubbles reduces the number of pores and the volume fraction of porosity (Figure 1h), as the trapped bubbles have several chances to vanish.^[^ ^1^, ^7^, ^29^ ^]^ Furthermore, there is a stabilization effect through the higher local speed of the keyhole itself and the melt flow is changing, too. In contrast to the static beam guidance the energy input is more widely distributed, which leads to smaller temperature gradients and less accumulation of hydrogen. Occasionally beam oscillations can also lead to gas concentrations in pinned single bubbles, squeezing them to elongated pores in the welding direction through the high dynamic movement of the keyhole (Figure 1e,f).

The detailed cut‐out in Figure 2c shows the keyhole shape and position in 3D as the laser beam advances. The fluctuation of the vapour capillary is a highly dynamic process and can only be represented to a limited extent in the static laser welding condition considering the available tomoscopic recording rate of 100 tps which corresponds to an integration over 10 ms for each tomogram. The keyhole center moves with 1 mmin^−1^, i.e., by ∼167μm within 10 ms, while the fluctuation of the capillary shape takes place in fractions of a millisecond.^[^ ^7^, ^30^, ^31^ ^]^ Through the superimposed 1 kHz oscillation, the absolute velocity of the beam rises to values of ∼331 mms^−1^, 20 times higher than the 16.7 mms^−1^ for static beam guidance, which prevents 3D reconstruction of the keyhole during beam oscillation. Nevertheless, the effect of the beam oscillation on pore formation and weld defects such as blow outs are clearly visible in both cases and confirm previous studies.^[^ ^1^, ^7^, ^29^, ^32^ ^]^

## 3 Conclusion

We studied the keyhole and bubbles in the melt pool in all three spatial directions during the welding process at a realistic technological rate of 1 mmin^−1^ for a conventional static and a dynamic beam guidance condition with a superimposed beam oscillation during welding of an aluminum alloy. We found that dynamic beam guidance with a frequency of 1 kHz reduced porosity by 75 % and improved seem surface roughness attributed to a reduced temperature gradient in the liquid weld seem due to a more distributed energy introduction and by the fast release of pinned large bubbles by the oscillating keyhole. This knowledge could help reduce defects in the welding of materials such as aluminum die‐casting alloys and enable targeted optimization of welding parameters, especially for multi‐parameter approaches such as dynamic beam modulation.

## 4 Experimental Section

### Tomoscopy Setup

By acquiring many X‐ray radiographs from different angles of view and reconstructing the 3D distribution of absorption coefficients mathematically, one can obtain a tomogram. Tomography avoids the ambiguities and misinterpretations of radiography caused by the superposition of features in one direction and the associated loss of information. Tomoscopy expresses time‐resolved tomography at high repetition rates and is sometimes also called 4D tomography. An intense white X‐ray beam was provided by a superconducting 2.9 T bending magnet at the TOmographic Microscopy and Coherent rAdiology experimenTs (TOMCAT) beamline of the Paul Scherrer Institute's Swiss Light Source, Villigen, Switzerland and filtered with 5 mm of glassy carbon and a 325μm‐thick Si wafer to reduce the low‐energy radiation impact to the system. The radiation transmitted through the sample was detected by a 150μm‐thick LuAG:Ce scintillator (Crytur, Czech Republic), and the visible image created projected through a 4× magnification high‐resolution macroscope (Optique Peter, France)^[^ ^33^ ^]^ onto the GigaFRoST camera system,^[^ ^34^ ^]^ which allows for fast recording for long times. The frame rate was 12 500 images per second, the effective pixel size 2.75μm (measured spatial resolution at 100 tps of 7.6μm)^[^ ^21^ ^]^ and the field of view 3 mm × 0.8 mm. The samples were placed in a graphite crucible mounted on the rotation stage and rotated with 50 Hz while tomograms were recorded every 180°, which leads to 100 tomograms in a second.

### Sample Preparation and Laser Welding Setup

**Figure 4:**
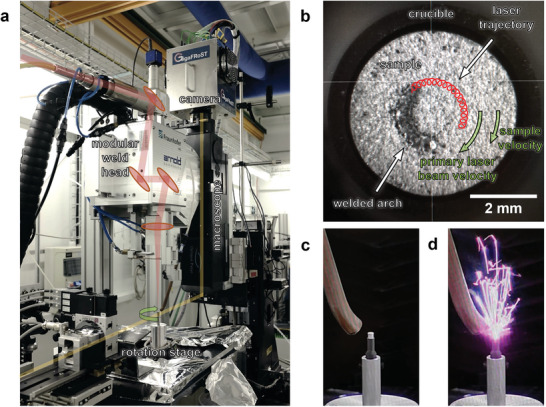
a) Welding and tomoscopic setup at TOMCAT beamline, with laser beam path marked in red and X‐ray beam path in yellow. b) Welded arch on the sample as seen by the camera in the laser head with dynamic beam modulation drawn in red with the number of turns reduced by a factor of 10. The direction of movement of the sample and the laser are indicated by green arrows, but are not represented in their actual proportion. c) Sample placed on a graphite crucible, which is mounted on the rotation stage. On the left is the supply line for inert gas. d) Set‐up from c) with laser operating and creating flying spatters.

Die‐cast aluminum alloy AlSi9Cu3(Fe) samples were used to study the laser welding process in operando. For this purpose, cylindrical samples of 5 mm diameter and 6 mm height were prepared out of 300 mm × 200 mm × 6 mm plates. A portable single‐mode fibre laser system (600 W max. power, 1070 nm wavelength) was used for the bead on plate welding experiments. It includes a system for rapid beam control (“remoweld®FLEX” welding optics) which was installed above the rotating sample (see **Figure** **4**). A slight flow of nitrogen (10 Lmin^−1^) as a shielding gas was blown over the sample to provide uniform conditions and reduce oxidation. The laser was operated at 400 W and focused onto the sample surface, yielding a spot of 33μm diameter. The quality/brilliance of the laser beam and the small spot enable a stable coupling of the beam into the material and are necessary for a successful usage of the dynamic beam modulation explained below. A circular movement of 1 mm radius of the laser beam concentrically aligned with the rotation axis of the tomoscopy setup was created using the programmable positioning system of the laser system. This movement allowed to produce a circular weld seam on the rotating sample advancing at a tangential speed of 1 mmin^−1^ relative to the requested tomographic rotation (50 Hz), while continuously recording tomograms at 100 tps. To complete a circle, the laser was operated for 377 ms, during which 38 tomograms were acquired. Additionally to the laser beam revolution, a superimposed oscillatory beam modulation during the feed motion is made possible by the integrated welding optics. In the studied case, a 2D circular oscillation with a frequency of 1 kHz and an amplitude of 0.05 mm was applied for dynamic beam guidance. More detailed information about the laser setup can be found in previous work.^[^ ^7^ ^]^

### Data analysis

Tomoscopy allows for operando time‐resolved imaging in 3D of the porosity formation and evolution during welding. Dynamic features and processes were studied with realistic welding parameters (laser velocity and power), whereby the sample size was adapted to the tomoscopy method, taking into account the X‐ray transmission and centrifugal forces. Segmented volumes were extracted from series of tomograms and time‐dependent quantitative analyses such as of porosity, number of pores, equivalent pore diameter distribution (calculated from the pore volume assuming a spherical shape), etc. performed and weighted to characteristic values such as the pore or weld volume. The shape parameter expressing pore elongation was determined via a principal component analysis of the voxels belonging to a pore and is described by 1 minus the ratio of the mean to the largest eigenvalue of their covariance matrix. If these variances are equal (as for spherical objects) a zero value is obtained, while elongated objects have a value approaching 1. After the welding process, a separate tomogram of each sample was recorded with 2000 projections over 0.5 s. Due to the higher contrast in the microstructure and its change after melting and solidification, the welds can be well distinguished and segmented from the base material by means of variance filters and morphological operations.

## Conflict of Interest

The authors declare no conflict of interest.

## Supporting information

Supporting Information

## Data Availability

The data that support the findings of this study are available from the corresponding author upon reasonable request.
