# Supplementary material for: Operando X‐Ray Tomoscopy of Laser Beam Welding
Source: Adv Sci (Weinh). 2025 Jan 13;12(9):2413108. doi: 10.1002/advs.202413108 (PMC11884564; doi:10.1002/advs.202413108)
Supplement: Supplementary file 1 — Supporting Information [file ADVS-12-2413108-s001.pdf]

ADVANCED SCIENCE
Open Access

Supporting Information

for *Adv. Sci.*, DOI 10.1002/advs.202413108

Operando X-Ray Tomoscopy of Laser Beam Welding

Paul Hans Kamm, Stephan Börner, Tillmann Robert Neu, Christian Matthias Schlepütz, Dirk Dittrich, John Banhart and Francisco García-Moreno*

Supporting Information

Operando X-ray Tomoscopy of Laser Beam Welding

Paul Hans Kamm, Stephan Börner, Tillmann Robert Neu, Christian Matthias Schlepütz, Dirk Dittrich, John Banhart, Francisco García-Moreno*

Considering the weld seam extent and rotation speed of the specimen, radial accelerations acting on the melt are 6 and 14 *g* for the inner and outer edge of the seam, respectively. Although the images do not show an obvious effect of centrifugal forces the distribution of the pores with respect to the centre of the welding circle does show a slight skew (**Figure S3**). A slight pore accumulation is seen more towards the inner side of the circular weld (62% or 60% of the pore volume of the statically or dynamically welded sample is located within the welding circle radius), which could be the effect of the melt dragged outwards by the centrifugal force of the rotating samples during the experiments. However, the small sample and welding circle size might also influence the process in terms of higher laser energy density, inferior heat dissipation and increased risk of heat accumulation on the inside, which in turn influence the dynamics of the melt and the gas and can also cause the skewed distribution in Figure S2.

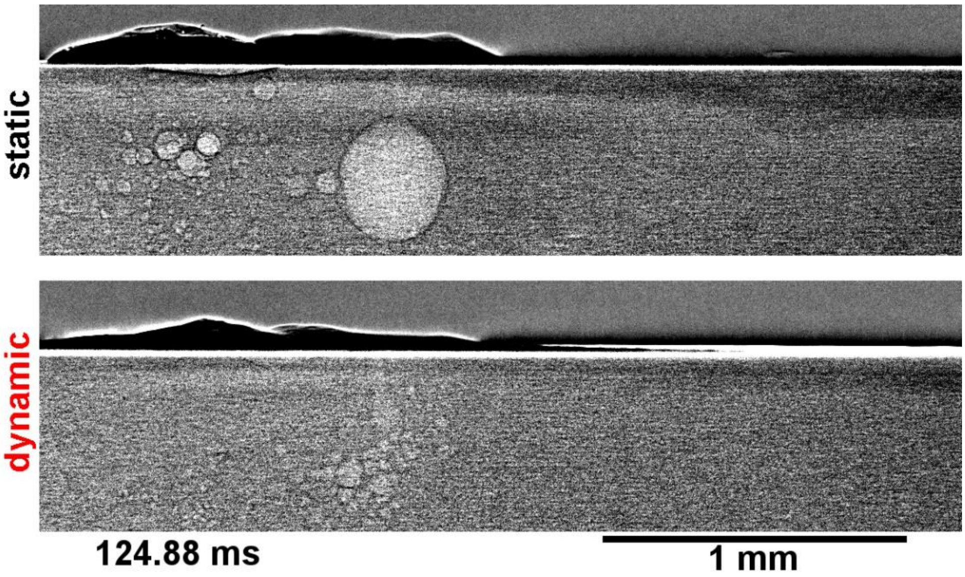

Video S1. Projection sequence (still image or downloadable here: http://tomoscopy.net/videos/videoS1.mp4) of a total of two rotations (four tomograms) during welding with linear/static (top) and dynamic (bottom) beam guidance.

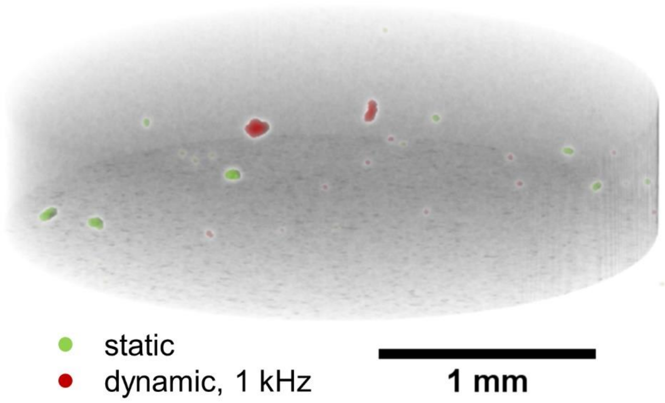

Figure S1. Superimposed 3D renderings of the specimens for the static (green) and dynamic (red) process before welding (first tomogram in series) with highlighted voids in corresponding colour (exaggerated representation by dilation with a spherical structural element with a diameter of 7 Px).

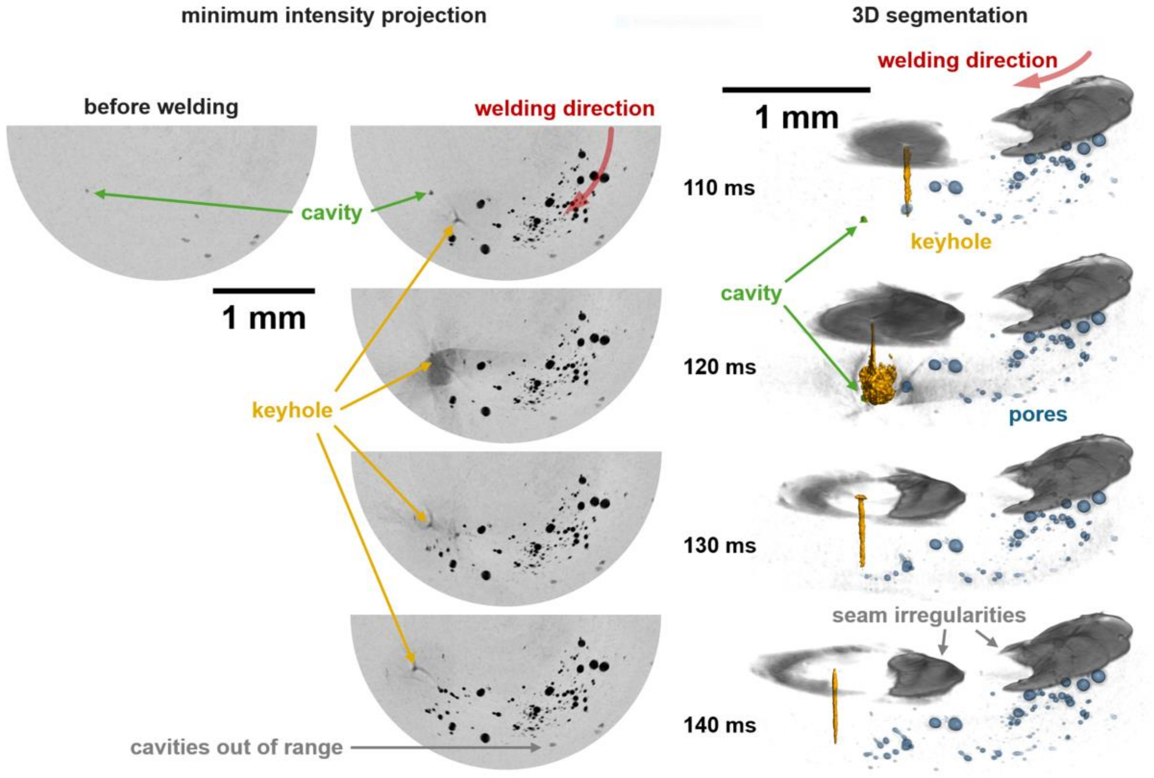

Figure S2. Minimum intensity projection (in vertical direction) of the virtually halved sample of the static process in the initial state (left) with existing cavities, at different time points (middle column from top to bottom) and with their corresponding 3D renderings (right column) showing the keyhole (orange), the passed over cavity (green), as well as the resulting pores (blue) and seam irregularities (grey) around the event from Figure 3c.

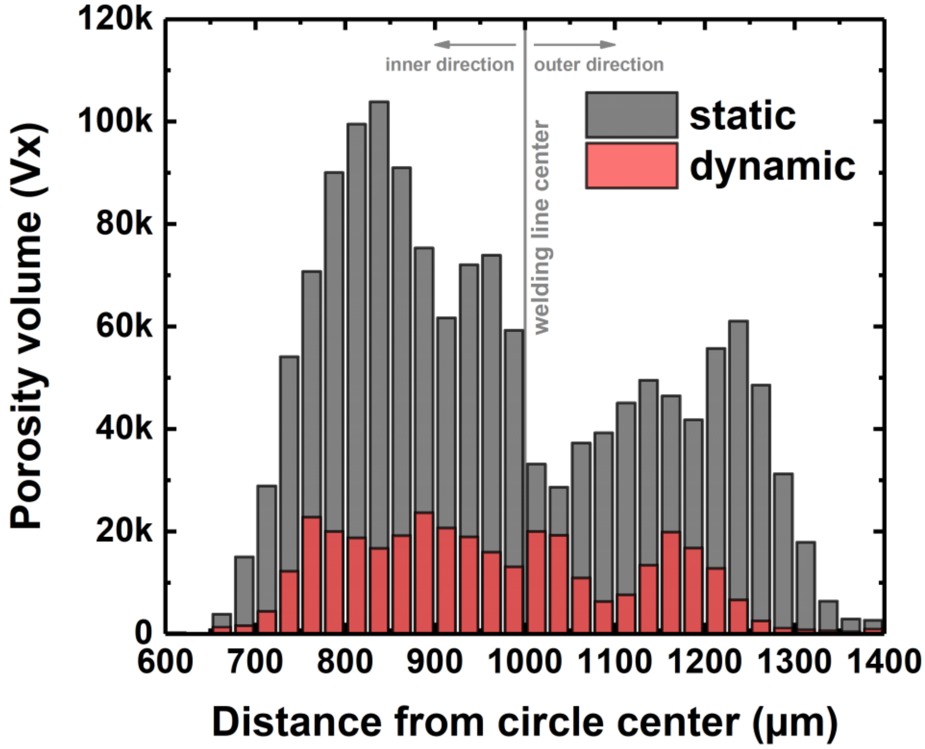

Figure S3. Distribution of the porosity volume as a function of the distance to the centre of the welding circle after welding (last tomogram in series) for the static (black) and dynamic case (red).
